# Editorial: Glial Cells, Maladaptive Plasticity, and Neurodegeneration: Mechanisms, Targeted Therapies, and Future Directions

**DOI:** 10.3389/fncel.2021.682524

**Published:** 2021-04-30

**Authors:** Sohaib Ali Korai, Giovanna Sepe, Livio Luongo, Andrea Beatriz Cragnolini, Giovanni Cirillo

**Affiliations:** ^1^Division of Human Anatomy – Laboratory of Neuronal Networks, University of Campania “Luigi Vanvitelli”, Naples, Italy; ^2^Division of Pharmacology, University of Campania “Luigi Vanvitelli”, Naples, Italy; ^3^IRCSS Neuromed, Pozzilli, Italy; ^4^Facultad de Ciencias Exactas, Físicas y Naturales, Universidad Nacional de Córdoba, Córdoba, Argentina; ^5^Instituto de Investigaciones Biológicas y Tecnológicas (IIByT), CONICET-Universidad Nacional de Córdoba, Córdoba, Argentina

**Keywords:** glial cells, neuroinflammation, neurodegeneration, synaptic homeostasis, synaptic plasticity

Understanding the biological complexity of the central nervous system (CNS) is a frontier in neuroscience. Morphological organization of the CNS represents the basis for its functional properties underlying higher brain functions; therefore, efforts are needed to boost the comprehension of the organization of the CNS, from the ultrastructural to the functional-networks level.

To date, two highly integrated and interconnected cellular networks substantiate the anatomo-functional organization of CNS: neurons and non-neuronal cells, namely glial cells.

Glial cells, including astrocytes, oligodendrocytes, and microglia, actively participate in many complex functions within the CNS (immunity surveillance and inflammatory response, metabolic and synaptic homeostasis, modulation of blood-brain barrier—BBB) (Volterra and Meldolesi, 2005). Moreover, interaction with the elements of the extracellular matrix (ECM), an active player for long-term plasticity and circuit maintenance, adds another level of complexity to the modern model of the synapse structure (tetrapartite synapse) (Song and Dityatev, 2018). Therefore, if on one hand glial cells allow adaptive synaptic plasticity of CNS in several physiological conditions modulating synaptic transmission, homeostasis, and neural pathways signaling, then on the other, when activated, they boost inflammatory response and perturb neuroglial interactions, synaptic circuitry, and plasticity. This new condition, called maladaptive synaptic plasticity, may represent an early stage of neuroinflammatory processes in neurodegenerative disorders (Papa et al., 2014).

Recently, it has been hypothesized that the morpho-functional heterogeneity of astrocytes in different brain regions might explain the regional diversity of astrocytic response to an external injury and the selectivity of neuronal degeneration (Cragnolini et al., 2018, 2020). Therefore, the comprehension of these mechanisms is relevant for the development of targeted therapies for clinical management of neurodegenerative disorders. Only through unraveling the complex interactions between the different cell types at the synapse, we will truly understand synaptic plasticity, higher brain functions, and how perturbations of these interactions contribute to brain diseases with dramatic clinical impact.

This Research Topic aims to address the role of neuroglia in health and disease through a collection of 12 articles that include two mini-reviews, two reviews, one hypothesis and theory, and seven original research articles, providing an overview on a broad spectrum of molecular and cellular bases of glial function in normal and pathological CNS.

New mechanisms were explored in glial transmission, signaling, and neural circuitry. It has been established that astrocytes which release neurotransmitters such as glutamate and GABA, express a variety of neurotransmitter receptors and participate in synaptic transmission (Araque et al., 2014). Furthermore, GABA released from interneurons participates in astrocyte-mediated control of excitatory synaptic transmission (Perea et al., 2016) through activation of astrocytic GABA-B receptors leading to gliotransmission (Liu et al., 2018b). The coupling between GABA interneuron, astrocytic terminals and pre/post synaptic compartments may underpin both seizure-like activity and neuronal synchrony across different brain regions (Liu et al.). Evidence has also demonstrated that early neuroinflammation, macrophage infiltration, and astrocytic activation have a main role in epileptogenesis (Rossi et al., 2013, 2017). In particular, experimental epileptic seizures after stroke, brain injury, or pilocarpine administration induce the release of small intracellular molecules (called DAMP—damage associated molecular patterns) including the ubiquitous High Mobility Group Box 1 (HMGB1) nuclear protein (Braun et al., 2017). It has been proposed that microglial-astroglial cooperation is required for astrocytes to respond to HMGB-1 and to induce neurodegeneration. Disruption of this HMGB-1 mediated signaling pathway shows beneficial effects by reducing neuroinflammation and neurodegeneration after status epilepticus (Rosciszewski et al.).

Furthermore, thrombin increase in the brain after stroke as a result of brain-blood barrier breakdown or brain tissue intrinsic production enhances PAR1-mediated NMDA receptor activity and increases intracellular calcium levels (Maggio et al., 2013). Altman et al. reviews the events that lead to a hyperexcitable state and to maladaptive plasticity that in turn, through an astrocytic mediated response, synchronize neuronal activity producing epileptic activity.

Astrocytic functions and activity are also affected by general anesthetics (Thrane et al., 2012; Liu et al., 2016), particularly the NMDA antagonist ketamine have an inhibitory role in astrocytic glutamatergic transmission blocking GluN1/GluN2B receptors. The decrease of astrocyte-mediated slow inward current (SICs) synchronization due to ketamine administration at clinical concentration may have serious implications for the development of dissociative cognitive impairment during ketamine anesthesia (Zhang et al.). In addition, anesthesia and surgical stress may cause postoperative cognitive dysfunction (POCD), a highly prevalent condition in elderly patients undergoing major surgery or non-invasive procedures under sedation (Leslie, 2017; Skvarc et al., 2018). Wei et al. hypothesized that mtROS/NLRP3 inflammasome may be part of the upstream pathway mediating the cleavage and release of the microglial interleukin 1 beta into brain areas including hippocampus that might play a pivotal role in POCD.

Besides their role in neural circuitry and signaling, glial cells are also involved in axonal regeneration and nerve repair. Ganglionic satellite glial cells (SGCs) create a structural unit inside the dorsal root ganglion, providing protection against inflammation, structural, and metabolic support to neurons (Hanani, 2005). Slit1 is one of the signaling factors that guides both axon projection and neuronal migration (Blockus and Chédotal, 2016), by mediating the branching of the sensory axon growth cone. Evidence has demonstrated that peripheral nerve injury activates the purinergic system via P2X7 receptors (mainly expressed on SGCs) and upregulates the expression of slit1, promoting axonal repair, and regeneration (Zhang et al.).

The relevance of astrocytes to the neurodegenerative process was highlighted in an experimental model of Parkinson's disease (PD). Astrocytes express the α7 nicotinic acetylcholine receptor (α7nAChR) (Xu et al., 2019) for anti-inflammatory (Egea et al., 2015), antiapoptotic (Kim et al., 2012), and neuroprotective functions in health and disease (Liu et al., 2015; Quik et al., 2015). The α7nAChR agonist PNU-282987 showed neuroprotective effects in astrocytes treated with 1-methyl-4-phenylpyridinium (MPP+), reducing the number of degenerating cells, and alleviating MPP -induced apoptosis. Moreover, PNU-282987 upregulated the expression of the antiapoptotic protein Bcl-2 and downregulated the expression of the apoptotic protein Bax and cleaved caspase-3, mainly via the JNK-p53-caspase-3 signaling (Hua et al.).

The immunological aspects of glial cells were explored by Zhou et al. in a murine model of experimental autoimmune encephalomyelitis (EAE). CD73, an astrocytic membrane ectonucleotidase, quickly converts adenosine monophosphate (AMP) to adenosine (an anti-inflammatory mediator) that interacts with ARA1 receptors controlling excitability, synaptic transmission in brain circuits and neuroinflammation (Liu et al., 2018a). Upon EAE induction, astrocytes lose most of their membrane-localized CD73, thus inhibiting the generation of adenosine in the local microenvironment, boosting the inflammatory process, and facilitating the pathogenesis of EAE (Zhou et al.).

Among the inflammatory disorders of the CNS, neuromyelitis optica spectrum disorder (NMOSD) represents a prototype of antigen-antibody-induced damage (the disease target is the aquaporin-4 (AQP4), a water channel protein expressed on astrocytic end-feet) (Wingerchuk et al., 2015). As in many other CNS disorders, glutamatergic excitotoxicity may play an important role in NMOSD (Zekeridou and Lennon, 2015). Da Silva et al. reviews the pathway that causes AQP4-IgG/AQP4 complex to downregulate the astrocytic glutamate transporter EAAT2 (Yang et al., 2016), leading to the excitotoxic damage.

Activation of glial cells, dysfunction of the endocannabinoid signaling and increased expression of pro-inflammatory factors might also contribute to pathogenesis of autism spectrum disorder (ASD) (Bronzuoli et al., 2018). Using the ASD animal model consisting in prenatal exposure to valproic acid (VPA) (Melancia et al., 2018), it has been demonstrated that an inflammatory reaction and astrocytic/microglia activation restricted to the hippocampus and related to impaired social interactions, stereotypic repetitive behaviors, and learning and memory defects. Interestingly, treatment with the phytocannabinoid cannabidivarin (CBDV) restores hippocampal endocannabinoid signaling and neuroinflammation induced by prenatal VPA exposure and recovered social impairments, social novelty preference, short-term memory deficits, and repetitive behaviors (Zamberletti et al.).

Connexins and pannexins are widely expressed in glial cells, where they play several roles including channel and non-channel functions. These functions include modulation of synaptic gain, the control of excitability through regulation of the ion and neurotransmitter composition of the extracellular milieu and the promotion of neuronal survival (Abudara et al., 2018). Brocardo et al. review how glial connexins and pannexins are remodeled in different pathological conditions with variable outcomes in the context of a neurodegenerative disease.

The established role of glial cells in many neurological and neuropsychiatric disorders has boosted new glial-targeted therapies, however the unavailability of validated biomarkers to assess and monitor gliosis *in vivo* has limited their clinical application (Garden and Campbell, 2016). Cavaliere et al. present a comprehensive overview on molecular neuroimaging, using positron emission tomography (PET) and magnetic resonance imaging (MRI) and offer a wide panel of non- or minimally invasive techniques to image glial targets.

Altogether, this Research Topic has provided an update on recent developments in glial biology, with many translational insights for a wider understanding of the neural mechanisms in health and disease.

## Author Contributions

All authors contributed in writing the editorial and organizing the research topic.

## Conflict of Interest

The authors declare that the research was conducted in the absence of any commercial or financial relationships that could be construed as a potential conflict of interest.
